# Diabetic Cataract in Spontaneously Diabetic Torii Fatty Rats

**DOI:** 10.1155/2020/3058547

**Published:** 2020-08-01

**Authors:** Kasumi Kikuchi, Miyuki Murata, Kousuke Noda, Satoru Kase, Yoshiaki Tagawa, Yasushi Kageyama, Masami Shinohara, Tomohiko Sasase, Susumu Ishida

**Affiliations:** ^1^Laboratory of Ocular Cell Biology & Visual Science, Japan; ^2^Department of Ophthalmology, Faculty of Medicine and Graduate School of Medicine, Hokkaido University, Sapporo, Japan; ^3^Tokyo Animal & Diet Department, CLEA Japan, Inc., Tokyo, Japan; ^4^Biological/Pharmacological Research Laboratories, Central Pharmaceutical Research Institute, Japan Tobacco Inc., Osaka, Japan

## Abstract

Spontaneously Diabetic Torii (SDT) fatty rat is a novel animal model of type 2 diabetes with obesity. SDT fatty rats develop hyperglycemia, dyslipidemia, and other diabetic complications including ocular disorders; however, diabetic cataract formation in SDT fatty rats has not been fully investigated. The aim of the current study was to investigate the characteristics of cataract in the SDT fatty rats. The mean body weight of SDT fatty rats is larger than that of age-matched Sprague-Dawley (SD) rats and control animals until 8 weeks of age, and thereafter the growing speed decreased until the end of observation at 16 weeks of age. Blood glucose levels in SDT fatty rats were significantly higher than those in SD rats throughout the observational period. Slit-lamp examination revealed that no rats showed cataract formation at 5 weeks of age; however, SDT fatty rats gradually developed cortical cataract and posterior subcapsular cataract, both of which are the common types of cataract in patients with type 2 diabetes. The levels of glucose, sorbitol, and fructose were higher in the lens tissues of SDT fatty rats in comparison with that of SD rats. Furthermore, the level of 4-hydroxynonenal (4-HNE) was higher in the lens of SDT fatty rats than in that of SD rats. By contrast, total glutathione (GSH) concentration was lower in the lens of SDT fatty rats than in that of SD rats. The present study demonstrated that the cataractogenesis in SDT fatty rats resembled human diabetic cataract formation, indicating that SDT fatty rats serve as a potential animal model in researches on human cataract associated with type 2 diabetes and obesity.

## 1. Introduction

The prevalence of diabetes is increasing worldwide, with an estimated global prevalence of 463 million in 2019, and with a projected upsurge affecting 578 million people by 2030 and 700 million people by 2045 [1]. Diabetes is associated with an increased risk of visual disturbance due to ocular complications including cataract, retinopathy, and neovascular glaucoma [2]. Among the aforementioned conditions, cataract is one of the major causes of legal blindness in patients with diabetes, as previously reported in the Wisconsin Epidemiological Study of Diabetic Retinopathy [3, 4]. Consequently, elucidation of the molecular mechanism underlying the cataractogenesis in diabetic patients, which requires extensive and efficient researches, may lead to the development of an effective pharmacological approach to alleviate the social burden.

Previous literature on human diabetic cataract has reported elevated glucose concentration [5] and subsequent increase in the levels of sorbitol and fructose [5, 6], both of which are generated through glucose metabolism in the polyol pathway. Furthermore, oxidative stress is known to play an instrumental role in the development of diabetic cataract [7, 8]. Moreover, a previous study has reported that the levels of reduced glutathione (GSH), an antioxidant peptide, were observed to be lower in human diabetic cataract than in cataract in age-matched, nondiabetic individuals [8]. The evidence from human subjects suggests that the increase in glucose uptake and the loss of antioxidant activity in the lens initiate the development of cataract in diabetic patients. Nevertheless, the detailed etiology of diabetic cataract remains elusive, and a suitable animal model is required to carry out further research on the development of cataract in diabetic patients.

The Spontaneously Diabetic Torii (SDT) fatty rat is a novel animal model of type 2 diabetes, which was established by introducing the fa allele of the leptin receptor gene of the Zucker fatty rat into the genome of the SDT rat, a former animal model of non-obese type 2 diabetes found in an inbred strain of Sprague-Dawley (SD) rat [9, 10]. SDT fatty rats develop diabetic conditions such as polyphagia, hyperglycemia, and dyslipidemia from the age of five weeks [11] and manifest diabetic complications from the age of about twenty weeks [12]. Although the ocular complications of diabetes have been reported in previous literature [11, 13, 14], no previous study has performed comprehensive researches on the development of cataract in SDT fatty rats.

The aim of this study was to investigate the characteristics of cataract in SDT fatty rats and to compare them with the characteristics of cataract in human diabetic patients to elucidate the similarities.

## 2. Materials and Methods

### 2.1. Animals

Male SDT fatty rats were kindly donated by the CLEA Japan, Inc. (Tokyo, Japan). As a control, age-matched male SD rats were used [14, 15]. The rodents were housed in the animal facility at Hokkaido University. Normal food and water were provided *ad libitum*. All animal experiments in the current study were conducted in accordance with the guidelines of the Association for Research in Vision and Ophthalmology (ARVO) Statement for the Use of Animals in Ophthalmic and Vision Research. The experiment was approved by the Ethics Review Committee for Animal Experimentation of the Hokkaido University (#18-0115).

### 2.2. Estimation of Body Weight, Blood Glucose, and Triglyceride Levels

During the course of the current study, all the rodents underwent weekly body weight and blood glucose estimations. Nonfasting blood glucose level was measured by means of tail blood samples using Glutest mint (Sanwa Kagaku, Aichi, Japan). The rats were euthanized through deep anesthesia at the ages of five, eight, twelve, and sixteen weeks. Subsequently, the chest cavity was opened and blood was collected from the right atrium. The collected blood was centrifuged at 1710 × g for 10 minutes at 4°C, and the serum was collected. Serum triglyceride was measured using a Serum Triglyceride Quantification Kit (Cell Biolabs, San Diego, CA).

### 2.3. Slit-Lamp Examination and Cataract Classification

The slit-lamp examination was performed on a weekly basis. Anesthesia was administered using 2.5% isoflurane; the pupils were dilated by means of topical tropicamide and phenylephrine hydrochloride and subsequently, the eyes were examined using a slit-lamp biomicroscope. Progression of lenticular opacity was unbiasedly graded into five stages by two ophthalmologists (KK and YT), as described in a previous literature: stage 0, normal lenses; stage 1, minimal opacity at the center of the lens; stage 2, patchy appearance of opacity, both in the center and periphery of the lens; stage 3, uniform opalescence all over the lens; stage 4, mature cataract with nuclear opacity [16, 17].

### 2.4. Determination of Glucose, Fructose, Sorbitol, Total GSH, and 4-HNE Levels in the Lens

All the rats were euthanized by means of anesthetic overdose, and the eyes were enucleated. Intact lenses were dissected and harvested through an anterior approach and stored at -80°C, until further examination. The lenses were homogenized and sonicated in 150 *μ*l of PBS. The solution was centrifuged at 17,700 × g for 10 minutes at 4°C, and the supernatants were collected. The protein concentration and 4-hydroxynonenal (HNE) levels in the supernatants were determined through the BCA protein assay (Thermo Fisher Scientific, Tokyo, Japan) and the OxiSelect 4-HNE adducts competitive ELISA kit (Cell Biolabs), respectively. After deproteination through 5% metaphosphoric acid treatment, the levels of glucose, fructose, sorbitol, and total GSH in the supernatants were measured using the Glucose-Glo assay (Promega, Madison, WI), PicoProbe fructose assay kit (BioVision, Milpitas, CA), EnzyChrom sorbitol assay kit (BioAssay Systems, Hayward, CA), and the total glutathione assay kit (Jaica, Shizuoka, Japan), respectively.

### 2.5. Histology

The enucleated eyes were fixed overnight using Super Fix KY-500 (Kurabo Industries Ltd., Osaka, Japan). Subsequently, the tissues were processed and embedded in paraffin using the standard techniques. The sections were stained using hematoxylin and eosin (H&E) and observed under a light microscope.

### 2.6. Statistical Analysis

All the results are expressed as mean ± standard error and the *n*-numbers are specified. Statistical analysis of the difference in mean values between the two groups was performed using the Student's *t*-test. The current study considered the difference to be statistically significant when the probability values were less than 0.05.

## 3. Results

### 3.1. Progression of Diabetes in SDT Fatty Rats

**Figure 1:**
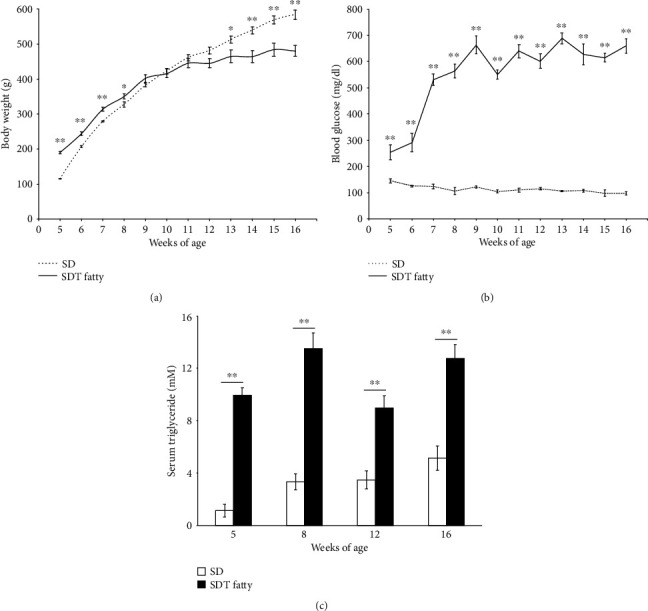
Systemic parameters of SDT fatty rats and SD rats. (a) Body weight. SDT fatty rats, *n* = 5 each; SD rats, *n* = 6 each. ^∗^*p* < 0.05; ^∗∗^*p* < 0.01. (b) Blood glucose level. SDT fatty rats, *n* = 5 each; SD rats, *n* = 6 each. ^∗∗^*p* < 0.01. (c) Serum triglyceride level. SDT fatty rats, *n* = 5 to 10; SD rats, *n* = 5 to 11. ^∗∗^*p* < 0.01.

The mean body weight of the SDT fatty rats (*n* = 5) was observed to be significantly higher than that of the SD rats (*n* = 6), until the age of eight weeks. Subsequently, the SDT rats exhibited a decline in the growth rate (Figure 1(a)). During the time period from the age of five weeks to sixteen weeks, the blood glucose levels in SDT fatty rats were observed to be significantly higher than those in the SD rats (Figure 1(b)). The serum triglyceride levels in SDT fatty rats were observed to be higher than those in the SD rats (Figure 1(c)).

### 3.2. Diabetic Cataract Formation in SDT Fatty Rats

**Figure 2:**
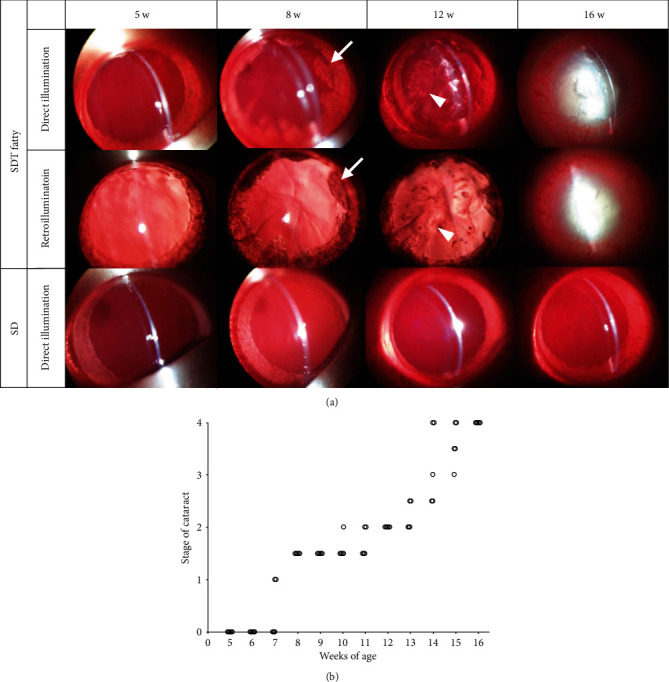
Time course of cataract formation in SDT fatty rats. (a) Representative images of cataract observed in SDT fatty rats using slit-lamp microscope. Arrows indicate cortical cataract formation. Arrowheads indicate posterior subcapsular cataract formation. At 16 weeks of age, SDT fatty rats developed mature cataract. By contrast, cataract formation was not observed in SD rats throughout the observational period. (b) Chronological observation of cataract progression in the identical animals. *n* = 5.

**Figure 3:**
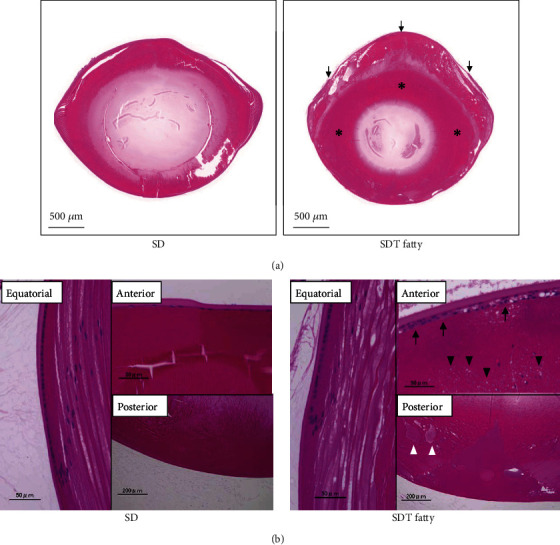
Histopathological findings in the lens tissues of SDT fatty rats and SD rats. Representative micrographs of the H&E stained lens extracted at 16 weeks of age. (a) Low magnification. Marked liquefaction beneath the anterior capsule (arrows), and eosinophilic amorphous lesions around the nucleus (asterisk) were observed in the lens of SDT fatty rats. (b) High magnification. Increased number of nuclei in epithelial cells (arrow), vacuolation (black arrowhead), and liquefaction (white arrowhead) were observed. Bar = 50 *μ*m.

In order to examine the progression of diabetic cataract in SDT fatty rats, the grade of lens opacity was evaluated by means of the slit-lamp examination. In SDT fatty rats, the onset of cataract formation was observed at the age of seven weeks. As a result, the SDT fatty rats gradually developed cortical cataract and posterior subcapsular cataract (PSC), both of which are characteristically seen in patients with type 2 diabetes, which eventually resulted in mature cataract formation (stage 4) at the age of 16 weeks. In contrast, lens opacification was not observed in SD rats during the course of the study (Figures 2(a) and 2(b)). Histological analysis of the lens revealed marked liquefaction beneath the anterior capsule, and eosinophilic amorphous lesions around the nucleus in SDT fatty rats, compared with SD rats (Figure 3(a)). In high magnification, there were the increased number of nuclei in the epithelial cells, vacuolation, and liquefaction, whereas no remarkable changes were observed in the lenses of SD rats (Figure 3(b)).

### 3.3. Polyol Pathway Products in the Lenses of SDT Fatty Rats

**Figure 4:**
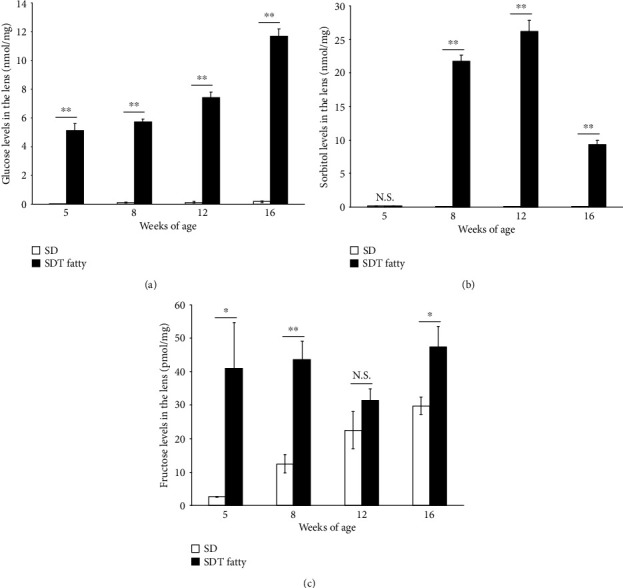
Sugar levels in the lens tissues of SDT fatty rats and SD rats. The levels of (a) glucose, (b) sorbitol, and (c) fructose in the lens tissues. SDT fatty rats, *n* = 5 to 10; SD rats, *n* = 5 to 11. N.S.: not significant; ^∗^*p* < 0.05; ^∗∗^*p* < 0.01.

The present study measured the glucose, sorbitol, and fructose levels in the lenses of SDT fatty rats, in order to verify the association between the polyol pathway and cataractogenesis. The current study observed that the glucose levels in the lenses of SDT fatty rats were significantly higher than those of the SD rats; assessed at the ages of eight weeks (5.74 ± 0.17 nmol/mg, *n* = 5, *p* < 0.01), twelve weeks (7.41 ± 0.40 nmol/mg, *n* = 5, *p* < 0.01), and sixteen weeks (11.69 ± 0.50 nmol/mg, *n* = 10, *p* < 0.01, Figure 4(a)). At the age of five weeks, the glucose level in the lenses of SD rats was below the detection limit of the assay. However, the glucose levels were observed to increase gradually during the course of the study (8 weeks, 0.12 ± 0.05 nmol/mg, *n* = 5; 12 weeks, 0.13 ± 0.06 nmol/mg, *n* = 5; 16 weeks, 0.18 ± 0.06 nmol/mg, *n* = 11).

At the age of five weeks, the sorbitol level in the lenses of SDT fatty rats was below the detection limit of the assay. However, the sorbitol levels elevated until 12 weeks (8 weeks, 21.72 ± 0.95 nmol/mg, *n* = 5; 12 weeks, 26.18 ± 1.71 nmol/mg, *n* = 5) and decreased at 16 weeks (9.31 ± 0.67 nmol/mg, *n* = 10; Figure 4(b)). In contrast, sorbitol was not detected in the lenses of SD rats during the course of the study (Figure 4(b)).

At the ages of eight and sixteen weeks, the fructose levels in the lenses of SDT fatty rats were observed to be significantly elevated (8 weeks, 43.58 ± 5.41 pmol/mg, *n* = 5, *p* < 0.01; 16 weeks, 47.31 ± 6.23 pmol/mg, *n* = 10, *p* < 0.05), compared to the SD rats (8 weeks, 12.52 ± 2.74 pmol/mg, *n* = 5; 16 weeks, 29.81 ± 2.52 pmol/mg, *n* = 11). At the age of five weeks, the fructose levels in the lenses of SD rats were below the detection limit of the assay. In comparison, the fructose levels were elevated in the lenses of SDT fatty rats (41.02 ± 13.68 pmol/mg, *n* = 5). The current study observed a tendency towards elevated fructose levels in the lenses of SDT fatty rats, compared to the SD rats, at the age of 12 weeks (SD, 22.56 ± 5.49 pmol/mg, *n* = 5; SDT fatty, 31.47 ± 3.46 pmol/mg, *n* = 5; Figure 4(c)).

### 3.4. Elevation of Oxidative Stress in the Lenses of SDT Fatty Rats

**Figure 5:**
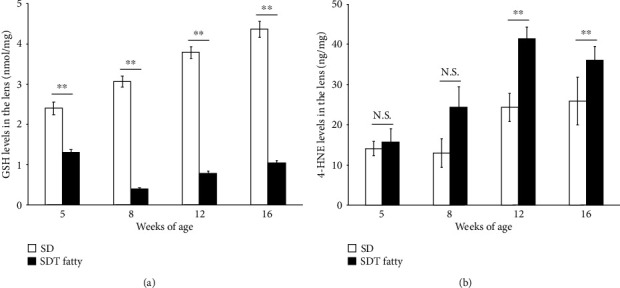
Elevation of oxidative stress in the lens tissues of SDT fatty rats and SD rats. The levels of (a) total GSH and (b) 4-HNE in the lens. SDT fatty rats, *n* = 5 to 10; SD rats, *n* = 5 to 11. N.S.: not significant; ^∗∗^*p* < 0.01.

The present study measured the levels of total GSH and an oxidative stress marker, 4-HNE, in order to evaluate the status of oxidative stress in the lens. The total GSH levels in the lenses of SDT fatty rats were observed to be significantly lower than those in the SD rats (5 to 12 weeks, *n* = 5 in each group; 16 weeks SD rats, *n* = 11; 16 weeks SDT fatty rats, *n* = 10; *p* < 0.01, Figure 5(a)). Moreover, a significant increase in 4-HNE levels was observed in the lenses of SDT fatty rats, compared to the SD rats, at the ages of 12 (SD rats, 24.35 ± 3.53 ng/mg, *n* = 5; SDT fatty rats, 41.38 ± 2.95 ng/mg, *n* = 5; *p* < 0.01) and 16 weeks (SD rats, 25.92 ± 5.94 ng/mg, *n* = 11; SDT fatty rats, 36.07 ± 3.38 g/mg, *n* = 10; *p* < 0.01) (Figure 5(b)).

## 4. Discussion

In the present study, cortical cataract and PSC were the main types of lens opacification in SDT fatty rats, both of which are the characteristic phenotypes of cataract in patients with type 2 diabetes. Furthermore, glucose, sorbitol, and fructose concentrations elevated in the lens of SDT fatty rats, consistent with previous studies on human diabetic cataract. Finally, reduced antioxidant GSH and elevated 4-HNE, an oxidative stress marker, were observed in the lens of SDT fatty rats. These findings indicate that SDT fatty rats serve as a spontaneous diabetic animal model in order to investigate the detailed molecular mechanisms of diabetic cataract formation.

Previous studies have demonstrated that SDT fatty rats represent the early-onset type of diabetic animal model. Diabetic characteristics were detected at the age of five weeks in males and eight weeks in females, with an incidence of 100% at the age of 16 weeks in males and 73% at the age of 32 weeks in females [10]. The body weight gain is higher in SDT fatty rats than control SD rats until the age of eight weeks; however, the growth rate of SDT fatty rats was gradually declined in this study, in common with the end stage of diabetes in humans. Previously, it was reported the SDT fatty rats rapidly showed sarcopenia, age-related decrease of muscle mass and strength, between the age of eight weeks and 16 weeks [15]. Therefore, it is likely that body weight loss observed in SDT fatty rats after the age of eight weeks is a consequence of sarcopenia. Furthermore, in accordance with the previous data, the present study demonstrated that SDT fatty rats manifest hyperglycemia and dyslipidemia at the age of five weeks and sustained the high glucose level throughout the observational period. In SDT fatty rats, cataract development was also studied by means of macroscopic observation and the tissue staining method [11, 18]. In addition to the previous histological analysis, the current study observed the temporal progression of cataract formation using the slit-lamp biomicroscope examination and revealed that SDT fatty rats developed cortical cataract and PSC within two months after birth. As aforementioned, cortical cataract and PSC are the common types of lens opacification seen in human diabetic cataract [19]. The morphological similarity in lens opacification indicates that SDT fatty rats and human diabetic patients share a common pathway of cataract formation. However, an increased number of nuclei in epithelial cells observed in SDT fatty rats was inconsistent with the previous finding reported as characteristics of human diabetic cataract [20]. The pathological discrepancy between SDT fatty rats and humans might be due to the different duration of cataract formation or species *per se*.

The present study demonstrated that the sugar levels were higher in the lens of SDT fatty rats, compared to SD rats. Previous literature has reported that the intracellular polyol pathway is activated in diabetes. Under hyperglycemic conditions, excess glucose enters the polyol pathway and is converted to sorbitol by aldose reductase, and subsequently, sorbitol is converted to fructose by sorbitol dehydrogenase. Consequently, sorbitol accumulates in the epithelial cells in the lens, resulting in osmotic pressure changes, lens fiber swelling, and, eventually, cataract formation [21]. Moreover, a previous study has reported increased concentrations of glucose, sorbitol, and fructose in the lens of diabetic patients [5]. The aforementioned study observed increased sorbitol levels in the lens of diabetic patients, while sorbitol was not detected in the lens of nondiabetic subjects [5]. In the current study, the sorbitol level gradually increased in the lens of SDT fatty rat at 8 and 12 weeks of age. Thereafter, the lens sorbitol level unexpectedly decreased at 16 weeks of age. A similar shift of the lens sorbitol level was also observed in streptozotocin- (STZ-) induced diabetic rats" because this is the first apperance [22]. One possible explanation for the common phenomenon found in diabetic rodent models is that the processing of excessive glucose via polyol pathway facilitated the consumption of cofactor NAPDH in the lens and resulted in the subsequent reduction of sorbitol generation by aldose reductase. NADPH is also known as a required cofactor for the function of glutathione reductase, which catalyzes the reduction of oxidized glutathione to GSH. Therefore, it is likely that consumption or depletion of NADPH caused by excessive glucose processing is also associated with an increase of oxidative stress in the lens.

Previous literature has established that oxidative stress plays a role in cataract formation. GSH contributes to the antioxidant system and maintains the transparency of the lens. Under conditions of oxidative stress, oxidative GSH nonenzymatically oxidizes the neighboring protein thiol to form protein-S-S-glutathione and successively, protein-protein disulfide, which promotes protein aggregation and subsequent lens opacity [23]. The present study observed decreased levels of total GSH from the early ages in the SDT fatty rats, and considerably increased levels of 4-HNE, an oxidative stress marker, at the age of 12 weeks, which displayed a slightly delayed onset, in comparison with early-onset reduction in total GSH levels. A previous study has reported that the 4-HNE levels were observed to be significantly increased in the aqueous humor obtained from patients with diabetic retinopathy [24]. The aforementioned biochemical analyses and the results indicate that SDT fatty rats and human diabetic patients share certain common features of cataract.

Previously, STZ-induced diabetes in rodents were commonly used as an animal model in researches on diabetic cataract [25–27]. STZ enters the cytoplasm of *β*-cells in the pancreas [28] via glucose transporter-2 and reduces insulin secretion through cell toxicity [29–31] and thus, the STZ-injected animals develop hyperglycemia, resembling the conditions seen in type I diabetes. In addition, the rodents with STZ-induced diabetes exhibit cataract formation, increased glucose accumulation [32], and decreased GSH [22] in the lens. However, the cataract in STZ-injected animals differs from the cataract seen in patients with type 2 diabetes in certain aspects. Primarily, the hyperglycemia in STZ-injected animals occurs due to acute pharmacological interruption of insulin secretion in the pancreas, which is completely different from the pathogenesis of type 2 diabetes, indicating that the molecular mechanism of lens opacification in the STZ animal model differs from that in type 2 diabetic humans. In fact, the STZ model directly develops the intumescent white cataract due to cortical lens fiber swelling [33], while cortical cataract and PSC are the characteristic forms of lens opacity in patients with type 2 diabetes, as mentioned previously. This implies that the selection of an animal model warrants careful consideration, based on the purpose of the particular study, especially in studies like preclinical studies on molecular mechanism.

The present study has several limitations. First, we measured nonfasting blood glucose levels to confirm hyperglycemia in SDT fatty rats. However, to further elucidate the relationship between diabetic status and cataractgenesis in this animal model, measurements of fasting blood glucose and HbA1c were required. Second, the advanced glycation end products (AGEs), involved in diabetic cataract formation, were not measured. In hyperglycemic conditions, sugars react nonenzymatically with proteins and produces glycated proteins, which are further converted into AGEs. *α*-Crystallin, a major lens protein, acts as a chaperone-like molecule preventing the protein aggregation and maintaining the lens transparency. AGEs disturb the protein structure and the chaperone-like activity of *α*-crystallin and thus lead to cataract formation [34]. Future studies involving the comprehensive measurement of AGEs are required.

## 5. Conclusion

In summary, the development of cataract in SDT fatty rats mimicked human diabetic cataract formation biomicroscopically, morphologically, and biochemically. Previously, aldose reductase inhibitors have shown excellent results in the treatment of cataracts in diabetic rat models [35]; however, the results could not be replicated in human clinical trials. This discrepancy may be attributed to the use of the STZ-induced diabetes model, which differs from human type 2 diabetes in pathogenesis. The characteristics of the cataract in SDT fatty rats are similar to cataract in humans. Hence, the SDT fatty rat may contribute to the development of effective therapeutic agents for the treatment of human cataract.

## Data Availability

The data used to support the findings of this study are included within the article.
